# Predictors of Medically Serious Suicide Attempts: A Case–Control Study in Patients Admitted to a General Hospital Over Eight Years

**DOI:** 10.62641/aep.v53i6.1971

**Published:** 2025-12-17

**Authors:** Roberto Sánchez-González, Elisa Marí-Cardona, Eila Monteagudo-Gimeno, Luis Pintor-Pérez

**Affiliations:** ^1^Department of Psychiatry, Institut de Salut Mental, Hospital del Mar, 08003 Barcelona, Spain; ^2^IMIM (Hospital del Mar Medical Research Institute), 08003 Barcelona, Spain; ^3^Centro de Investigación Biomédica en Red de Salud Mental, Instituto de Salud Carlos III, 28029 Madrid, Spain; ^4^Faculty of Medicine and Life Sciences, Universitat Pompeu Fabra (UPF), 08003 Barcelona, Spain; ^5^Benito Menni Mental Health Care Complex, Sant Boi de Llobregat, 08830 Barcelona, Spain; ^6^Consultation-Liaison Service, Department of Psychiatry, Institut de Neurociències, Hospital Clínic i Provincial de Barcelona, 08011 Barcelona, Spain

**Keywords:** suicide attempt, psychiatry, suicidal behavior, risk factors, referral, case-control study

## Abstract

**Background and Objectives:**

A medically serious suicide attempt (MSSA) has been defined as a suicide attempt that would be fatal if not for medical intervention. Despite the seriousness of MSSA, the risk factors are only partially understood. The main aims of the present study were to define the characteristics of patients admitted to a general hospital for MSSA and to identify the predictors of MSSA.

**Methods:**

Prospective, observational case-control study involving adult inpatients admitted to non-psychiatric units at our institution and consecutively referred to the consultation-liaison psychiatry (CLP) unit between January 1, 2011 and December 31, 2018. Cases were patients who met clinical criteria for MSSA and controls were patients referred to the CLP unit for any other reason. All participants underwent a structured psychiatric interview. Sociodemographic, clinical, and psychosocial data were collected. Univariate and multivariate analyses were performed. Variables that were statistically significant on the univariate analysis were entered into a multivariate binomial logistic regression model.

**Results:**

A total of 5428 patients were included: 223 (4.1%) cases and 5205 (95.9%) controls. On the multivariate analysis, the variables significantly associated with the risk of MSSA were: younger age (odds ratio [OR] = 0.98); history of previous suicide attempts (OR = 17.41): psychosocial stressors (interpersonal problems, OR = 2.33; legal problems, OR = 3.38; multiple stressors, OR = 2.28); and presence of severe mental illness (schizophrenia, OR = 6.32; mood disorder, OR = 6.77; personality disorder, OR = 6.35).

**Conclusions:**

The findings of this study highlight the importance of early identification of individuals who present with risk factors for MSSA to enable timely intervention. The prompt intervention of CLP services plays a key role in improving patient outcomes, underscoring the importance of specialized, comprehensive psychiatric care in this patient population.

## Introduction

According to data from the World Health Organization (WHO) [1], approximately 727,000 people around the world died from suicide in 2021. Although suicide can occur at any age, it is more common in younger people, being the third leading cause of death worldwide among adolescents and young adults age 15–29 years in 2021 [1]. Given that suicide is a preventable cause of death, there is a clear need to detect early warning signs and to develop optimal strategies to prevent it.

The term “suicidal behavior” includes a wide range of behaviors, ranging from ideation and planning to non-suicidal self-injury, suicide attempts, and finally, completed suicide. The term “serious suicide attempt” (SSA) refers to an act that would have been lethal had it not been for intervention or chance, and/or that involve methods associated with a high probability of death [2]. Several different terms have been used to describe suicide attempts (SA) requiring hospital admission, including “near-fatal deliberate self-harm”, “near-lethal SA”, and “medically serious suicide attempt” (MSSA). Of these, the most widely accepted term is probably MSSA [3].

Numerous risk factors have been associated with SSAs, including younger age, psychiatric diagnosis, previous SA, and stressful psychosocial situations [4, 5, 6]. Other risk factors that have been associated with near-lethal suicide are severe tobacco dependence, mental pain, anxious and avoidant attachment patterns, and interpersonal difficulties [7, 8, 9]. However, relatively few studies have been performed to identify the risk factors specifically associated with MSSA. Similarly, the sociodemographic and clinical characteristics of this patient population and underlying risk factors have not been well characterized.

Suicidal behavior is a common reason for referral to consultation-liaison psychiatry (CLP) in general hospitals. In this regard, liaison psychiatrists play an important role as part of the multidisciplinary approach for the management of MSSA and prevention of recurrences [10].

In this context, the main aims of the present study were to (1) describe the characteristics of patients admitted to a general hospital for MSSA and treated by the CLP unit, and (2) identify predictive risk factors for MSSA. A secondary aim was to determine whether sex is a predictor of MSSA. We hypothesized that this clinical subgroup of suicide attempters would be associated with specific epidemiological and clinical features. The detection of risk populations may be useful to develop appropriate preventive measures.

## Materials and Methods

### Study Design

This was an observational case–control study conducted at the Hospital Clínic de Barcelona (Barcelona, Spain). Data were collected prospectively on consecutive inpatient consultation requests to our CLP unit over an 8-year period (January 1, 2011 to December 31, 2018).

### Setting

The Hospital Clínic de Barcelona is an 819-bed tertiary care general hospital with a catchment area of approximately 540,000 inhabitants. The adult CLP unit is staffed by two psychiatrists, a psychologist, and a psychiatric nurse.

### Participants

A non-probability sampling method was used for patient selection, which included all adults admitted to non-psychiatric units at our hospital who were consecutively referred to the CLP service. A total of 5428 consecutive inpatient consultation requests to our CLP service met the inclusion criteria.

Cases were selected according to the criteria established by Levi-Belz and Beautrais [2] and Beautrais [3]. A structured psychiatric interview was performed. All patients ≥18 years of age who met the following SA-related criteria were included as cases: (1) required hospital admission >24 hours; (2) met at least one of the following treatment criteria: (a) treatment in a specialized unit; (b) required surgery (superficial cuts excluded); (c) required extensive medical treatment (including antidotes for drug overdoses, telemetry, or repeated tests or studies); (d) used a highly lethal suicide method with a high risk of fatality (especially hanging or gunshot).

The control group was comprised of patients referred from medical or surgical units to the CLP service for any reason other than a suicide attempt.

To address potential sources of bias, we defined the following exclusion criteria: (1) only one referral for each admission (duplicate referrals for the same patient were excluded); (2) incomplete or inconsistent medical records; (3) admission to the psychiatric inpatient unit or discharge from the emergency department.

### Data Sources and Variables

All patients admitted to the hospital for more than 24 hours and referred to the CLP unit during the study period underwent a structured psychiatric interview. Most study-related data were collected directly from the patients during the interview. All other data were obtained from the patient’s medical history, hospital records, or from the referring physician, daily reports from the nurses, family members and/or caregivers.

The following variables were collected through the structured interview, an *ad hoc* questionnaire, and a prospectively compiled clinical database:

- Sociodemographic factors: age, sex, and physical disability.
- Psychiatric morbidity according to the Diagnostic and Statistical Manual of Mental Disorders, Fourth Edition, Text Revision (DSM-IV-TR) categories following administration of the Spanish version of Structured Clinical Interview for DSM-IV (SCID-CV) [11, 12]. The SCID is a semi-structured interview guide for making diagnoses according to the diagnostic criteria published by the American Psychiatric Association. The interview is administered by a clinician or trained mental health professional who is familiar with the DSM classification and diagnostic criteria.
- Psychiatric history, including any previous suicide attempts.
- Psychosocial stressors and exposure to recent stressful life events.
- Variables related to current hospitalization and CLP intervention: number of CLP visits; psychopharmacological intervention; length of hospital stay; and destination after hospital discharge.

### Statistical Methods

The principal clinical variables were characterized descriptively using absolute and relative frequencies (percentages) for categorical variables. Quantitative variables (e.g., age, length of stay) were described as means with standard deviation (SD). For categorical variables, the chi-square test was used to compare cases and controls. Distribution normality was checked with the Kolmogorov–Smirnov test. The Mann–Whitney U-test or Student’s *T*-test were used, as appropriate, to compare quantitative variables.

All variables that were statistically significant on the univariate analysis were entered into a multivariate, binomial logistic regression model, which included MSSA-related variables.

The Z-test for independent proportions was used to compare categorical variables related to current hospitalization and CLP intervention in the two groups. The regression model allowed us to control for the possible influence of confounders and to estimate their effects on the outcomes.

An analysis was performed to determine whether there were any differences between males and females within each subgroup (cases vs. controls).

The cut-off for statistical significance was set at *p* < 0.05 with 95% confidence intervals (CI). The IBM SPSS Statistics 28 program (Armonk, NY, USA: IBM Inc.) for Microsoft 365 was used to perform the statistical analyses.

## Results

### Descriptive Analysis of the Sample and Univariate Analysis

**Fig. 1.**
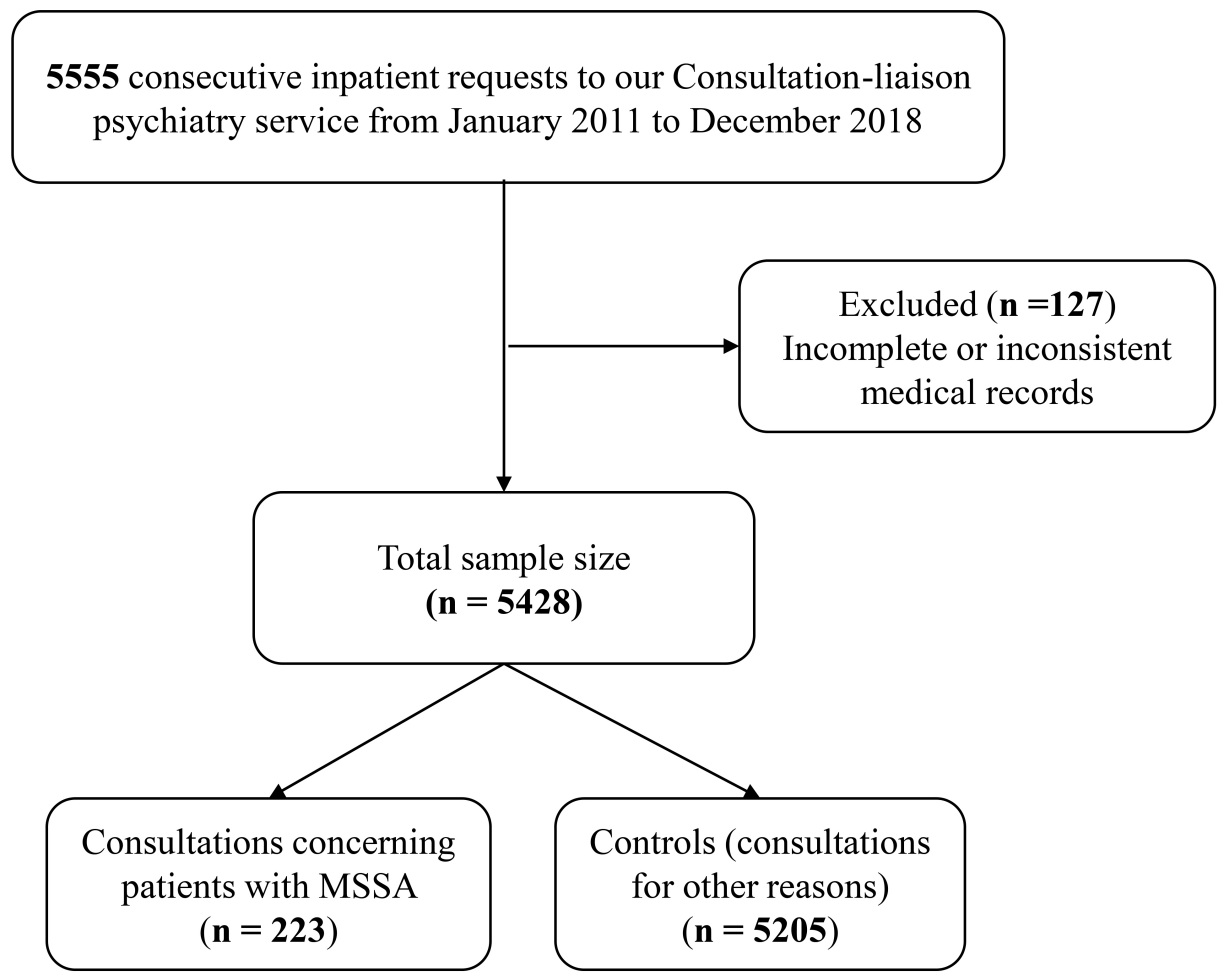
**Study flowchart**. MSSA, Medically serious suicide attempt.

The study flowchart is shown in Fig. 1. A total of 5428 patients were included; of these, 223 (4.1%) were cases and 5205 (95.9%) were controls. A total of 127 patients were excluded for incomplete or inconsistent medical records. There were no statistically significant clinical differences between the excluded patients and the controls.

The MSSA group was significantly younger than the controls, with a mean (SD) age of 47.3 (±19.4) vs. 55.3 (±17.6) years (*T*-test: 6.56, *p* < 0.001). Table 1 shows the results of the univariate analyses comparing the two groups.

**Table 1.** **Univariate analysis of sociodemographic and clinical variables**.

		Control group		MSSA group		X-Squared	*p* value
		(n = 5205)		(n = 223)			
		n	%	n	%		
Sex						1.74	0.167
	Male	2930	56.3	115	51.6		
	Female	2275	43.7	108	48.4		
Previous suicide attempts						1135.59	<0.001
	No	5046	96.9	102	45.7		
	Yes	159	3.1	121	54.3		
Psychiatric history						50.79	<0.001
	No	2368	45.5	47	21.1		
	Yes	2837	54.5	176	78.9		
Physical disability						0.29	0.585
	No	3847	73.9	169	75.8		
	Yes	1358	26.1	54	24.2		
Psychosocial stressors						102.19	<0.001
	None	3956	76.0	102	45.7		
	Financial	307	5.9	17	7.6		
	Interpersonal problems	668	12.8	72	32.3		
	Legal problems	33	0.6	7	3.1		
	Employment problems	81	1.6	8	3.6		
	Multiple stressors	160	3.1	17	7.6		
Current psychiatric diagnosis						343.63	<0.001
	Schizophrenia and/or other	198	3.8	28	12.5		
	psychotic disorders						
	Mood disorders	421	8.1	64	28.6		
	Adjustment disorders	1166	22.4	35	15.7		
	Personality disorders	146	2.8	42	18.9		
	Substance-related disorders	1254	24.1	14	6.4		
	Delirium, dementia, and cognitive disorders	1192	22.9	25	11.1		
	Other diagnosis	328	6.3	10	4.5		
	No diagnosis	500	9.6	5	2.3		

Abbreviations: MSSA, medically serious suicide attempt.

On the univariate analysis (Table 1), the variables significantly associated with MSSA were: age; psychiatric history; previous SA; psychosocial stressors; and presence of current psychiatric morbidity according to DSM-IV-TR. As Table 1 shows, the MSSA group was significantly younger, with a higher prevalence of previous psychiatric history, previous suicide attempts, and psychosocial stressors. Psychiatric diagnoses were more prevalent in the MSSA group, mainly severe mental illnesses (schizophrenia and other psychotic disorders, mood disorders, and personality disorders). By contrast, adjustment disorders, substance-related disorders and delirium/dementia were more prevalent in the control group. There was no association between sex or physical disability and a diagnosis of MSSA.

### Multivariate Analysis: Sociodemographic and Clinical Predictors of MSSA

Table 2 shows the results of the multivariate binomial logistic regression analysis.

**Table 2.** **Multivariate binomial logistic regression model showing predictors of MSSA adjusted for sociodemographic and clinical variables**.

		B	SE	OR	95% CI	*p* value
Age		–0.019	0.005	0.981	0.972–0.991	<0.001
Previous suicide attempt		2.857	0.187	17.414	12.062–25.143	<0.001
Psychiatric history		–0.158	0.220	0.854	0.555–1.313	0.472
Psychosocial stressors						
	Economic	0.583	0.313	1.791	0.969–3.309	0.063
	Interpersonal problems	0.847	0.193	2.334	1.597–3.410	<0.001
	Legal problems	1.219	0.591	3.382	1.062–10.775	0.039
	Employment-related problems	0.492	0.460	1.635	0.663–4.031	0.285
	Multiple stressors	0.824	0.345	2.279	1.160–4.477	0.017
Current psychiatric diagnosis						
	Schizophrenia and other	1.844	0.416	6.323	2.799–14.282	<0.001
	psychotic disorders					
	Mood disorder	1.912	0.376	6.769	3.240–14.145	<0.001
	Adjustment disorder	0.814	0.366	2.256	1.101–4.625	0.026
	Personality disorder	1.849	0.412	6.355	2.835–14.244	<0.001
	Substance-related disorder	–0.055	0.446	0.946	0.395–2.266	0.901
	Delirium, dementia, cognitive disorder	0.759	0.393	2.136	0.989–4.614	0.053
	Other diagnosis	0.523	0.476	1.687	0.663–4.292	0.272

Abbreviations: B, regression coefficient; CI, confidence interval; OR, odds ratio; SE, standard error.

As that table shows, the risk of MSSA was lower in older patients (OR = 0.98, 95% CI = 0.972–0.991, *p* < 0.001) and higher in patients with history of previous suicide attempts (OR = 17.41, 95% CI: 12.062–25.143, *p* < 0.001), psychosocial stressors (interpersonal problems, OR = 2.33, 95% CI: 1.597–3.410, *p* < 0.001; legal problems, OR = 3.38, 95% CI: 1.062–10.775, *p* = 0.039; or multiple stressors, OR = 2.28, 95% CI: 1.160–4.477, *p* = 0.017), and in patients with a severe mental illness (schizophrenia, OR = 6.32, 95% CI: 2.799–14.282, *p* < 0.001; mood disorders, OR = 6.77, 95% CI: 3.240–14.145, *p* < 0.001; personality disorders, OR = 6.35, 95% CI: 2.835–14.244, *p* < 0.001).

### Sex-Stratified Comparative Analysis

In the MSSA group, a sex-stratified comparative analysis revealed between-group difference (Table 3). In both sexes, the strongest predictors of MSSA were previous SA and current psychiatric diagnosis. However, among males, the psychiatric diagnosis associated with the highest risk of MSSA was mood disorder (OR = 5.795, 95% CI: 2.275–14.762, *p* < 0.001) followed by schizophrenia and other psychotic disorders (OR = 4.171, 95% CI: 1.481–11.747, *p* < 0.007). By contrast, among females, the psychiatric diagnoses associated with the greatest risk of MSSA were personality disorders (OR = 18.069, 95% CI: 4.684–69.706, *p* < 0.001), mood disorders (OR = 11.234, 95% CI: 3.065–41.183, *p* < 0.001), schizophrenia (OR = 10.045, 95% CI: 2.376–42.459, *p* = 0.002), and adjustment disorders (OR = 3.975, 95% CI: 1.127–14.022, *p *= 0.032).

**Table 3.** **Gender-stratified multivariate binomial logistic regression model showing predictors of MSSA**.

		Male					Female				
		B	SE	OR	95% CI	*p* value	B	SE	OR	95% CI	*p* value
Age		–0.022	0.007	0.978	0.964–0.992	0.002	–0.017	0.007	0.983	0.969–0.997	0.014
Previous suicide attempt		3.152	0.270	23.373	13.764–39.691	<0.001	2.603	0.270	13.505	7.960–22.911	<0.001
Psychiatric history		–0.050	0.305	0.951	0.523–1.730	0.869	–0.288	0.325	0.750	0.396–1.418	0.376
Psychosocial stressors											
	Economic	0.174	0.477	1.190	0.467–3.029	0.716	1.102	0.429	3.009	1.298–6.973	0.010
	Interpersonal problems	1.045	0.277	2.844	1.652–4.897	<0.001	0.672	0.275	1.958	1.142–3.357	0.015
	Legal problems	1.037	0.734	2.821	0.669–11.885	0.158	2.036	1.254	7.659	0.656–89.388	0.104
	Problems related to employment	0.371	0.618	1.449	0.431–4.866	0.549	0.595	0.707	1.814	0.454–7.250	0.400
	Multiple stressors	0.641	0.502	1.898	0.709–5.077	0.202	1.117	0.500	3.057	1.148–8.137	0.025
Current psychiatric diagnosis											
	Schizophrenia and other	1.428	0.528	4.171	1.481–11.747	0.007	2.307	0.735	10.045	2.376–42.459	0.002
	psychotic disorders										
	Mood disorder	1.757	0.477	5.795	2.275–14.762	<0.001	2.419	0.663	11.234	3.065–41.183	<0.001
	Adjustment disorder	0.480	0.476	1.616	0.636–4.107	0.313	1.380	0.643	3.975	1.127–14.022	0.032
	Personality disorder	1.050	0.566	2.858	0.942–8.664	0.064	2.894	0.689	18.069	4.684–69.706	<0.001
	Substance-related disorders	–0.293	0.534	0.746	0.262–2.125	0.583	0.169	0.871	1.184	0.215–6.526	0.846
	Delirium, dementia, and cognitive disorders	0.439	0.484	1.551	0.601–4.005	0.364	1.252	0.711	3.497	0.869–14.080	0.078
	Other diagnosis	0.056	0.733	1.057	0.251–4.445	0.939	1.264	0.733	3.541	0.843–14.883	0.084

Abbreviations: B, regression coefficient; CI, confidence interval; OR, odds ratio; SE, standard error.

### Analysis of Variables Related to Current Hospitalization and CLP Intervention for MSSA

We performed a *post hoc* comparative analysis to assess clinical differences between the MSSA and control groups during hospitalization and the CLP specific interventions for each subgroup (Table 4).

**Table 4.** **Comparison of patients and controls in hospitalization and CLP-related variables**.

Variable		MSSA group	Control group	*p* value
		%		
Number of follow-up visits at CLP service				
	≥2	77.1	57.8	*p * < 0.05
	1	22.9	42.2	
Psychopharmacological agents				
	Antipsychotics	36.8	29.1	*p * < 0.05
	Antidepressants	35.4	26.3	*p * < 0.05
	Mood stabilizers	4.7	2.2	*p * < 0.05
	Benzodiazepines	7.1	17.4	*p * < 0.05
	Non-psychopharmacological treatment	15.6	21.7	*p * < 0.05
Mean (SD) length of hospital stay, days		18.4 (17.4)	25.3 (17.9)	*p * < 0.003
Percentage of patients requiring an extension of stay at psychiatric inpatient unit		24.3	1.6	*p * < 0.05
Additional psychiatric health care indicated (community psychiatric care)		51.0	25.6	*p * < 0.05

Abbreviations: CLP, consultation-liaison psychiatry; SD, standard deviation.

As Table 4 shows, there significant differences between the groups in hospitalization and CLP-related variables. A significantly higher proportion of the MSSA group required ≥2 follow-up visits at the CLP and treatment with antipsychotics, antidepressants, and mood stabilizers. The mean hospital length of stay was shorter in the MSSA group, although 24.3% of these patients required extended hospitalization in the psychiatric inpatient unit, versus only 1.6% of controls.

## Discussion

The main objectives of this study were to describe the sociodemographic and clinical characteristics of patients hospitalized for MSSA and to identify predictors of risk. We included a large sample (n = 223) of patients with MSSA treated at our CLP unit over an 8-year period. On the multivariate analysis, several variables were significant risk factors for MSSA, including younger age, history of a previous suicide attempt, psychosocial stressors, and severe mental illness. Neither sex nor physical disability was associated with MSSA.

### Age

In our study, age was one of the four risk factors for MSSA. This finding is largely in line with previous studies that have shown that suicide attempts—which are infrequent during childhood and puberty—increase in early adulthood but then tend to decrease after age 55 [13, 14, 15]. Studies have shown that there are 100–200 suicide attempts for every completed suicide in the 15–24-year age group, highlighting the importance of prevention in young adults [13].

The risk of a second suicide attempt has been found to increase substantially when the first attempt occurred at a young age, making this subgroup of patients particularly susceptible to MSSA and/or a completed suicide [14]. The higher risk in young adults can be explained, in part, by social pressure, an increase in major life events, lack of access to mental health care, substance use, and family or relationship conflicts [13]. In this population, addressing the underlying risk factors and providing accessible mental health support could play a key role in preventing MSSA and completed suicide.

### History of Previous Suicide Attempts

In our sample, a history of previous suicide attempts was present in 121 of the 223 (54.3%) MSSA cases, which is consistent with previous studies showing this variable to be among the greatest risk factors for SA, MSSA, and completed suicide [14]. This finding was statistically significant in both males and females, making it one of the first factors to consider when stratifying patients for MSSA risk.

This association between previous SA and completed suicides has been observed in many studies. A review by Hawton and van Heeringen [15], showed that 40% of suicide victims had previously attempted suicide. Favril* et al*. [16] conducted a systematic review and metanalysis (37 studies from 23 countries) to evaluate the risk factors associated with suicide attempts. That review identified several risk factors, most notably the presence of an existing mental or personality disorder and certain sociodemographic factors (isolation, unemployment). In the meta-analysis, the two most relevant risk factors were a previous SA and a history of self-harm.

### Psychosocial Stressors

We observed a significant association between psychosocial stressors (e.g., financial problems, interpersonal problems, legal problems, or multiple stressors) with the risk of MSSA (Table 2). However, we found no association between physical disability risk of MSSA, probably because of the similar prevalence rates in cases and controls (24.2% and 26.1%, respectively).

The association between psychosocial stressors and suicide risk is well known. According to data from the WHO [17], nearly three-fourths (73%) of suicides in 2021 occurred in low- and middle-income countries. One clear example of the impact of stressors on suicide risk is the rise of unemployment and economic hardship that occurred in Greece during the post-2008 economic crisis. During that time period (2008–2014), there was a notable increase in suicidal ideation and suicide attempts, which demonstrates the impact an unstable socio-economic climate can have on mental health [18]. Similarly, as McMahon* et al*. [19] have demonstrated, psychosocial and psychiatric issues are highly prevalent before completed suicides. In that study, the authors found that at least one adverse event was present in the personal life of victims in the one-year period prior to death [19]. They also found that suicide attempts were more common in people who were single, unemployed, and/or living alone compared to controls. Similarly, some studies have shown that suicide attempts and suicidal ideation are higher among people who work in high stress environments (such as healthcare employees) [20].

As we have shown in this study, psychosocial stressors have a clear, negative impact on the risk of MSSA. However, our data suggest that there are important sex-related differences. In male patients, the only statistically significant stressor was interpersonal problems. By contrast, in females, interpersonal problems, financial problems, and multiple stressors were all associated with an increased risk of MSSA.

The aforementioned study by McMahon* et al*. [19] underscores the impact of socioeconomic instability and personal difficulties on suicide risk, emphasizing the need to implement targeted preventive measures. Our findings are consistent with those reported by McMahon and colleagues [19], thus providing further support for the association between psychosocial stressors and an increased risk of MSSA.

### Severe Mental Illness (Schizophrenia, Mood Disorders, Personality Disorders)

In the MSSA group, the most common risk factor was the presence of a current psychiatric diagnosis. The most prevalent of these were mood disorders, personality disorders, adjustment disorders, and schizophrenia or other psychotic disorders. Major depressive disorder (MDD) is also associated with an increased risk of suicide [21]. Bipolar disorder has been associated with a higher risk of death by suicide, especially during the depressive phase, making it one of the psychiatric conditions most closely linked to suicide [22].

Kim* et al*. [23] found that schizophrenia and other psychotic disorders were associated with serious suicide attempts with high medical severity, thus establishing schizophrenia as a specific risk factor for MSSA. In the literature, nearly every type of mental illness has been associated with an increased risk of SA [24]. Data from our study show that schizophrenia, mood disorders, and personality disorders are all significant risk factors.

### Sex

Richardson* et al*. [25] showed that suicide attempts are more common in females in almost every country of the world, even though males commit more completed suicides. This situation, known as the *gender paradox*, was first described by Canetto and Sakinofsky in 1998 [26]. Those authors demonstrated that males have a higher risk of completed suicide while being female is considered a risk factor for suicide attempts [21]. Notwithstanding those findings, we did not find any significant differences between males and females in terms of risk of MSSA. However, when stratified the study groups by sex, we did observe differences in other risk factors, particularly psychosocial stressors, which were significant risk factors in women but not in men. Similarly, the presence of a current psychiatric diagnosis (e.g., adjustment and personality disorders) was associated with a greater risk of MSSA in women (Table 3). The lack of association between this factor and suicide risk has been demonstrated by Park* et al*. [27].

### Management of MSSA by CLP Services

CLP units are comprised of dedicated psychiatry teams, generally based in general hospitals, whose role is to assess and treat mental health problems in the emergency department and hospital wards. CLP units provide integrated care of both physical and mental health problems to improve clinical outcomes. Several reviews have confirmed the value of hospital-based CLP services in terms of reducing the length of stay and higher patient satisfaction rates [10, 28].

In our study, patients with MSSA referred to the CLP unit had a significantly shorter length of stay in specialized units than controls, but a longer stay in the psychiatric unit, with a greater need for psychopharmacological treatment. Slightly more than half of MSSA patients (51%) were referred to psychiatric follow up through community mental health services versus only 25.6% of controls. These results are consistent with the study conducted by Bronson* et al*. [29], who concluded that CLP services add value to the healthcare system, potentially reducing the length of stay by 1.82 days and thus making the CLP a highly cost-effective alternative to traditional on-demand psychiatric consultations. Moretti* et al*. [30] also found that CLP is beneficial in general hospitals due to its multidisciplinary approach. Based on our findings, together with those reported in the available literature, it seems clear that patients referred to the CLP unit for MSSA require more intensive psychiatric care than those referred for other reasons.

### Limitations

The main limitations of this study are the single-center study design (external validity) and the non-random sampling technique (potential for bias), both of which limit the generalizability of our findings. Moreover, key study-related data were obtained directly from patients during structured interviews, which were conducted during a time of high stress. Consequently, some data related to the patients’ medical history and psychosocial stressors (among other data points) could be incomplete or biased. Another potential limitation is that the SCID interview was the only clinical scale administered. Other factors, such as the wide variability in mental health care settings and/or differences in CLP services in different countries, may reduce the generalizability of our results. Another limitation is that, at present, there are no standardized tools for diagnosing MSSA, which hinders our capacity to establish a clear separation between “serious” from “non-serious” suicide attempts. Moreover, the definition of “serious” can vary across hospitals and even among health care professionals, thus creating a potential source of bias. Despite these limitations, an important strength of this study is that it is the first to assess the risk factors for MSSA in a hospital setting with the involvement of a CLP unit.

## Conclusions

This study identified several significant risk factors for MSSA, including younger age, a history of previous suicide attempts, psychosocial stressors—especially interpersonal and legal problems—and severe mental illness (most notably, schizophrenia and mood/personality disorders). These findings show that patients with MSSA share several common characteristics, which could be used to help identify at-risk individuals to prevent new suicide attempts.

In the hospital setting, these patients have complex needs that are best addressed by experienced psychiatric and medical teams. In this regard, the availability of a clinical-liaison psychiatry unit within the hospital could potentially lead to better clinical outcomes in this patient population.

## Availability of Data and Materials

The data and materials that support the findings of this study are available from the corresponding author upon reasonable request.
